# Diagnostic Accuracy of the Forns Score for Liver Cirrhosis in Patients With Chronic Viral Hepatitis

**DOI:** 10.7759/cureus.14477

**Published:** 2021-04-13

**Authors:** Tayyaba Bukhari, Lena Jafri, Hafsa Majid, Sibtain Ahmed, Aysha Habib H Khan, Shahab Abid, Aniqa Raza, Imran Siddiqui

**Affiliations:** 1 Pathology and Laboratory Medicine, Aga Khan University Hospital, Karachi, PAK; 2 Gastroenterology, Aga Khan University Hospital, Karachi, PAK

**Keywords:** liver, fibroscan, cirrhosis, hepatitis b, hepatitis c, bilirubin, forns score

## Abstract

Introduction

Liver cirrhosis is an irreversible and end-stage disease. It results from chronic liver damage characterized by the replacement of normal liver tissue by fibrosis, leading to the progressive loss of liver function. Making an early diagnosis of cirrhosis is important for patients with chronic hepatitis because early antiviral therapy can prevent the progression of cirrhosis and even induce regression. There have been efforts to develop surrogate markers for liver cirrhosis as the biopsy is invasive, costly, and difficult to standardize.

Methods

This was a cross-sectional study conducted at the Section of Chemical Pathology, the Department of Pathology and Laboratory Medicine in Collaboration with the Section of Gastroenterology, Department of Medicine, the Aga Khan University, from January to December 2018. A total of 90 patients (>18 years of age) with a history of chronic viral hepatitis, who were attending the FibroScan® (Echosens, Paris, France) clinic were included. Patients with a history of autoimmune liver diseases and hepatocellular carcinoma were excluded from the study. Blood samples withdrawn were analyzed on ADVIA Centaur^® ^(Siemens Healthineers, Erlangen, Germany), and Forns scores were calculated based on the following four parameters: patient age, total cholesterol, gamma-glutamyl transferase (GGT), and platelet count.

Results

The median age of the patients was 38.5 years [interquartile range (IQR): 21]. Among the study population, 59 (65.6%) were males and 31 (34.4%) were females; 26 patients showed reactivity for hepatitis B surface antigen (HBsAg), and 63 patients were found chronic with hepatitis C virus (HCV). The proportion of HCV was observed to be higher as compared with that of Hepatitis B virus (HBV). Nineteen patients were found to have jaundice and only one patient had ascites. An Area Under the Receiver Operating Curve (AUROC) was generated to determine the diagnostic accuracy of the Forns score. It was observed that the Forn score value of >7.110 had an AUROC of 0.9928 (95% CI: 0.9821-1.003, p-value: <0.001) with a sensitivity of 100% (95% CI: 91.19-100.0%) and specificity of 94% (95% CI: 83.45-98.75%), with a higher positive likelihood ratio of 16.67.

Conclusion

This study found the Forns score to be sensitive and specific in diagnosing liver cirrhosis in patients with chronic hepatitis. The Forns score at a cutoff of 7.11 is highly sensitive as well as a specific noninvasive method that can be used to ascertain the status of fibrosis in chronic hepatitis patients.

## Introduction

Liver cirrhosis is an irreversible and end-stage disease if underlying etiology is not treated in the early stages. It is caused by chronic liver damage characterized by the replacement of normal liver tissue by fibrosis, leading to the progressive loss of liver function [1]. The global prevalence of liver cirrhosis is around 1-2% [2,3]. The estimated one-year mortality rate is 1-57% depending on the disease stage [4]. The most common causes of liver cirrhosis are alcohol-induced, non-alcoholic fatty liver disease (NAFLD), and chronic viral hepatitis with 10-20% of the patients with chronic hepatitis C and 12-20% of the patients with chronic hepatitis B progressing to liver cirrhosis worldwide [5,6]. Cirrhosis due to hepatitis C causes 0.5 million deaths every year [7,8].

Liver cirrhosis is one of the leading causes of mortality and frequent hospital admissions in developing countries like Pakistan [9]. One of the major causes is the high prevalence of viral hepatitis in our population, along with a poor healthcare system, failure to screen blood before transfusion, unhygienic clinical practices, poor infrastructure for infectious waste disposal, and limited access to healthcare facilities [10]. All these factors lead to a high prevalence of chronic viral hepatitis progressing to liver cirrhosis, with 41-52% cases with hepatitis C virus (HCV) and 30% with hepatitis B virus (HBV) [2]. Making an early diagnosis of cirrhosis is important for patients with chronic hepatitis because early and prompt initiation of antiviral therapy can prevent the progression of cirrhosis and even induce regression [11,12].

Liver biopsy has remained the gold standard for diagnosis in liver fibrosis for years [13,14]. However, there have been efforts to develop surrogate markers for liver cirrhosis as the biopsy is invasive, costly, difficult to standardize, and is contraindicated in patients with bleeding disorders, coagulopathy, and ascites [15]. Several researchers have used a noninvasive biochemical scoring tool known as the Forns score globally. The Forns score is calculated based on four parameters that include patient age, total cholesterol, gamma-glutamyl transferase (GGT), and platelet count. It is useful as a baseline determination of liver fibrosis; for monitoring changes in fibrosis over time: before, during, and after therapy or life-style modification; and as an aid in determining the prognosis [16]. The current study aimed to assess the diagnostic accuracy of the Forns score in identifying liver cirrhosis in patients of chronic viral hepatitis by taking FibroScan® (Echosens, Paris, France) as the benchmark.

## Materials and methods

This was a cross-sectional study conducted from January to December 2018 among patients with chronic viral hepatitis who were attending the FibroScan clinic at the Aga Khan University Hospital in Karachi, Pakistan. The study was conducted after obtaining ethical approval from the Ethical Review Committee of the Aga Khan University (ERC # Pat 5105). All patients (≥18 years of age) attending the FibroScan clinic were screened, and those patients with chronic liver disease related to ethanol abuse/NAFLD/autoimmune liver diseases/other common etiologies were excluded; patients with only viral hepatitis who agreed to sign the informed consent were included in the study. Clinical and biochemical details were collected via a structured questionnaire. The following biochemical parameters were noted: serum aspartate aminotransferase (AST), alanine transaminase (ALT), albumin, bilirubin, and serological markers for hepatitis [hepatitis B surface antigen (HBsAg) and antibodies to hepatitis C (anti-HCV)]. After receiving informed consent, 10 ml of patients’ blood was drawn in serum separator tubes and ethylenediaminetetraacetic acid (EDTA) for serum cholesterol, GGT, and platelet analysis. The samples were centrifuged at 3,000 RPM for 10-15 minutes. Serum cholesterol and GGT were analyzed on a spectrophotometer using kits by Merck (Merck & Co., Kenilworth, NJ) while platelet estimation was done on Sysmex XL 5000 (Sysmex Corporation, Kobe, Japan). The Forns score was calculated based on the following four parameters: patient age, total cholesterol, GGT, and platelet count. The Forns score equation used was as follows: [7.811 - 3.131 × ln [number of platelets] × 0.781 ln [GGTP (U/L)] + 3.467 × ln [age (years)] - 0.014 [cholesterol (mg/dL)]\]. The Forns score is unit-less [16].

Statistical analysis

SPSS Statistics version 21 (IBM, Armonk, NY) was used for data analysis. Medians with interquartile ranges (IQR) were reported for skewed data, which included age (years), cholesterol (mg/dl), GGT (U/L), platelet count (per microliter), FibroScan results, total alkaline phosphate (IU/L), ALT (IU/L), AST (IU/L), albumin (g/dL), bilirubin total (mg/dL), and direct and indirect bilirubin (mg/dL). Frequencies and percentages were computed for qualitative variables. The diagnostic accuracy of the Forns score with different cutoff points was assessed by using the Area Under the Receiver Operating Curve (AUROC), which was reported on a graph to show the sensitivity and specificity of the test. The overall accuracy of the test was assessed by measuring the sensitivity, specificity, positive predictive value (PPV), and negative predictive value (NPV). All analysis was done by a two-sided test with a 5% level of significance.

## Results

A total of 90 patients who met the inclusion criteria were included in this study. The median age of the patients was 38.5 years with an IQR of 21 years. In the study population, 59 (65.6%) were males and 31 (34.4%) were females. The majority had chronic hepatitis due to hepatitis C (anti-HCV reactivity) while 42.4% showed HBsAg reactivity. The proportion of HCV was observed to be higher as compared with that of HBV. Nineteen patients were found to have jaundice, while two had hepatomegaly and only one patient had ascites.

Median (IQR) values of the biochemical parameters of the study subjects are presented in Table 1.

**Table 1:** Biochemical and radiological parameters of study subjects (n=90) with liver cirrhosis due to chronic viral hepatitis IQR: interquartile range

Laboratory parameters	Median (IQR)
Cholesterol (mg/dL)	111.5 (66)
Gamma-glutamyl trasferase (U/L)	34 (63)
Platelet count (per microliter)	120 (230)
Total alkaline phosphatase (IU/L)	110 (47)
Alanine transaminase (IU/L)	48 (47.75)
Aspartate aminotransferase (IU/L)	39 (25)
Bilirubin total (mg/dL)	0.55 (0.5)
Direct bilirubin (mg/dL)	0.2 (0.2)
Indirect bilirubin (mg/dL)	0.3 (0.2)
Albumin (g/dL)	4 (1.05)
FibroScan (kPa)	6.85 (6.3)

**Figure 1:**
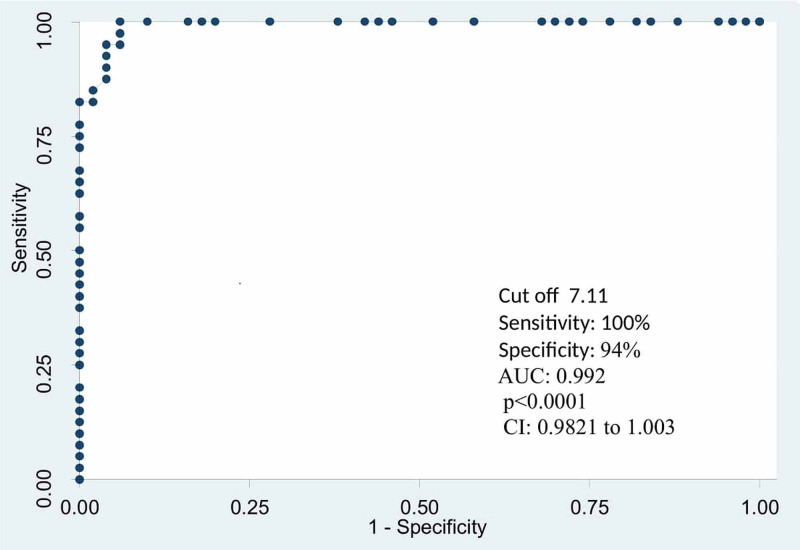
Receiver-operating characteristic curve of Forns score with a cutoff of 7.11 in diagnosing the presence of liver cirrhosis against liver FibroScan findings

The GGT levels were within the reference range in 36.7% (n=33) males as compared to females. Almost a majority of the male patients were observed to have normal ALP (49.9%; n=44), while ALT and AST levels were raised in all patients among both genders. Figure 1 shows an AUROC that was generated to determine the diagnostic accuracy of the Forns score.

It was observed that the Forns score value of >7.11 had an AUROC of 0.992 (95% CI: 0.9821-1.003, p-value: <0.001) with a sensitivity of 100% (95% CI: 91.19-100.0%) and specificity of 94% (95% CI: 83.45-98.75%).

## Discussion

Liver cirrhosis is considered to be one of the leading causes of mortality and frequent hospital admissions in developing countries [9]. In this current study, 70% of the study subjects suffered from liver cirrhosis due to hepatitis C infection, whereas 30% had it due to hepatitis B. This is in agreement with the study by Stefanescu et al. [17], where the most common cause of liver cirrhosis (49.78%) was chronic hepatitis C infection. An early diagnosis of cirrhosis is important for patients with chronic hepatitis because early and prompt initiation of antiviral therapy can prevent the progression of cirrhosis and even induce regression [11,12]. Liver biopsy is the gold standard in assessing the status of fibrosis in patients with liver disease [18]. However, liver biopsy has certain limitations, as it is invasive, painful, costly, and ineffective in providing details on mild liver diseases, especially those with a viral etiology [19].

To avoid the complications of invasive procedures, clinicians and investigators have been in search of noninvasive biochemical investigations or indices with high sensitivity and specificity. Since most of the chronic hepatitis patients are diagnosed at an early fibrosis stage, with mild fibrotic tissue on liver biopsy not revealing many details, using a noninvasive approach is much needed to avoid invasive procedures. Several investigators have studied many biomarkers to find an accurate serological marker for liver fibrosis [17]. Several serological markers and imaging techniques have been studied by using platelet count [20], ALT/AST ratio, prothrombin index, hyaluronate, macroglobulin, and haptoglobin to correctly identify HCV-infected patients with liver fibrosis [21].

FibroScan is an imaging technique that can be a good alternative to liver biopsy in the routine assessment of significant fibrosis [13,14]. Hence, in this study, FibroScan was taken as a benchmark for comparison with the Forns score, which is a noninvasive scoring method used for the assessment of liver cirrhosis. The prediction accuracy depicted by the Forns score for significant fibrosis was noted to be between 50 and 85% in a study [18]. This scoring system was initially validated in HCV patients. Controversy still exists regarding the cutoff values of the Forns score for diagnosing liver fibrosis in chronic liver disease patients. A cutoff value of 4.2 was chosen to identify the presence of liver fibrosis by Forns et al. initially [18]. By applying this cutoff, 45% of patients were identified with fibrosis, whereas, in other studies with a cutoff value of 6.9 for significant fibrosis, 96% of patients were correctly labeled [22,23]. In another study by Stefanescu et al., a Forns score cutoff of 7.3 was found with an AUROC of 0.648 for the diagnosis of liver cirrhosis [17]. In our study, a Forns score of >7.11 had an AUROC of 0.9928 (95% CI: 0.9821-1.003, p-value: <0.001*) with 100% sensitivity and 94% specificity.

There are certain limitations to this study. Firstly, it was a single-center study with a small sample size in which the viral load with the genotype of the subjects studied was not known. Secondly, it was not a longitudinal study; the progression of the disease and its association with the Forns score was not studied. The liver biopsy was not taken as the gold standard to calculate the diagnostic accuracy of the Forns score.

## Conclusions

This study found that the Forns score is a sensitive and specific noninvasive method that can be used to ascertain the status of fibrosis in chronic hepatitis patients. However, it has been found that most of the noninvasive markers are inconclusive in patients with early fibrosis. Hence, further studies are required including patients with other etiologies of fibrosis and cirrhosis as well. We also recommend comparing noninvasive markers with liver biopsy and correlating it with the grades of fibrosis in our population.
